# Linking Injury to Outcome in Acute Kidney Injury: A Matter of Sensitivity

**DOI:** 10.1371/journal.pone.0062691

**Published:** 2013-04-23

**Authors:** John W. Pickering, Zoltan H. Endre

**Affiliations:** 1 Christchurch Kidney Research Group, Department of Medicine, School of Medicine and Health Sciences, Otago University, Christchurch, New Zealand; 2 Department of Nephrology, Prince of Wales Clinical School, University of New South Wales, Sydney, Australia; University of Sao Paulo Medical School, Brazil

## Abstract

Current consensus definitions of Acute Kidney Injury (AKI) utilise thresholds of change in serum or plasma creatinine and urine output. Biomarkers of renal injury have been validated against these definitions. These biomarkers have also been shown to be independently associated with mortality and need for dialysis. For AKI definitions to include these structural biomarkers, there is a need for an independent outcome against which to judge both markers of functional change and structural markers of injury. We illustrate how sensitivity to need for dialysis and death can be used to link functional and structural (biomarker) based definitions of AKI. We demonstrated the methodology in a representative cohort of critically ill patients, in which an increase of plasma creatinine of >26.4 µmol/L in 48 hours or >50% in 7 days (Functional-AKI) had a sensitivity of 62% for death or dialysis within 30 days. In a development sub-cohort the urinary neutrophil-gelatinase-associated-lipocalin threshold with a 62% sensitivity for death or dialysis was 140 ng/ml (Structural-AKI). Using these thresholds in a validation sub-cohort, the risk of death or dialysis relative to those with no AKI by either definition was, for combined Structural-AKI and Functional-AKI 3.11 (95% Confidence interval: 2.53 to 3.55), for those with Structural-AKI but not Functional-AKI 1.51 (1.26 to 1.62), and for those with Functional-AKI but not Structural-AKI 1.34 (1.16 to 1.42). Linking functional and structural biomarkers via sensitivity for death and dialysis is a viable method by which to define thresholds for novel biomarkers of AKI.

## Introduction

AKI is common and associated with increased in-hospital mortality, length of stay and subsequent development of chronic kidney disease [1], [2]. The absence of symptoms to herald AKI mandates the monitoring of biomarkers for diagnosis. Current consensus definitions utilise an increase in plasma creatinine and a reduction in urine output as surrogates of a reduction in glomerular filtration rate [3]–[6]. Injury biomarkers detect AKI up to 48-hours earlier than creatinine [7] and offer a ray of sunshine in the dark and largely negative history of prevention and treatment of AKI [8]. Higher injury biomarker concentrations are associated with increasing AKI functional severity class and hard outcomes [9]–[11]. It has also been demonstrated, in the case of the injury biomarker, neutrophil-gelatinase-associated-lipocalin (NGAL), that biomarker-positive creatinine-negative patients have increased need for dialysis and higher mortality than patients negative for both creatinine and NGAL [12].

A major limitation in the evaluation of injury biomarkers is that performance is usually only assessed by the ability to detect or predict increases in plasma creatinine, the same parameter used to diagnose AKI [13]. Thus, while an increase in biomarkers can predict an increase in creatinine, only the inevitably delayed change in creatinine can diagnose AKI. This ignores that creatinine is a marker of function, while injury biomarkers detect structural damage. This strategy is analogous to using a functional marker, such as echocardiography, to diagnose myocardial infarction instead of a biomarker of injury, such as troponin. It also questions whether an increase in biomarkers alone should be used to facilitate early intervention, and thus argues against the advantages for which novel biomarkers have been developed. Finally, it ignores that the current consensus creatinine-based definitions of AKI have selected thresholds, creating dichotomous outcomes (AKI or no-AKI) from a continuous variable.

Although we do not yet know how much structural damage is required to reduce GFR acutely, we need an independent outcome against which to judge both markers of functional change and structural injury. The outcomes on which consensus is most likely are the requirement for dialysis and short-term mortality. While only dialysis links easily to AKI, there is increasing support for both direct and indirect linkage to death [14], [15]. However, even with large multicentre cohorts to assess these outcomes, there is no objective strategy for validating the functional or biomarker performance thresholds required to demonstrate clinical utility and to avoid the circular arguments inherent in the current approach. Choosing dialysis alone as the outcome would precipitate such a circular argument given that in clinical practice dialysis inevitably follows a substantial increase in plasma creatinine and therefore 100% sensitivity for the AKI threshold. In this scenario there would be no Structural-AKI without Functional-AKI with the outcome effectively benchmarking the structural biomarker once more against a functional definition of AKI.

If structural biomarkers are to have clinical utility, then a suitable methodology needs to be developed to determine a clinically relevant threshold. We hypothesised that sensitivity for a significant outcome (in this case, the composite of dialysis and death) is the ideal parameter for defining the threshold required for diagnosis of AKI by either a single structural or functional biomarker or for any combination of structural or functional biomarkers. We suggest that sensitivity for dialysis and death could provide the benchmark against which further enhancements should be judged. The use of sensitivity provides an independent parameter, to link these potentially disparate biomarkers. We chose, by way of example, to assess this methodology for a single candidate biomarker, urinary NGAL, by retrospective analysis of the well-documented dataset from the EARLYARF (Early Acute Renal Failure) trial. However, the method can equally be applied using any other candidate biomarker for structural injury in AKI.

## Materials and Methods

Patients from the EARLYARF trial in two intensive care units [16], [17] were randomly divided into development and internal validation cohorts stratified by AKI using the function-based Kidney Disease, Improving Global Outcomes (KDIGO) definition (Functional-AKI: an increase of plasma creatinine of either >26.4 µmol/L (0.3 mg/dl) in 48 hours or >50% in 7 days).

All biomarkers were measured at entry to ICU, and then at 12 and 24 hours. The maximum concentration for each patient was used in the analysis. Details of analysis have been reported previously [16], [17]. In brief, samples for alkaline phosphatase (AP), γ-glutamyl transpeptidase (GGT) were assayed immediately, by g-glutamyl-p- nitroanilide rate and p-nitrophenol rate reactions respectively (International Federation of Clinical Chemistry method). Samples for other assays were stored at −80°C until batch analysis. Urinary NGAL was measured using a NGAL ELISA Kit 036 (AntibodyShop, Grusbakken, Denmark) [18], Cystatin C (CysC) was measured with a BNII nephelometer (Dade Behring GmbH, Marburg, Germany) by particle-enhanced immunonephelometric assay [19], interleukin-18 (IL-18) using a human IL-18 ELISA kit (Medical and Biological Laboratories, Nagoya, Japan; see [20]), Kidney injury molecule-1 (KIM-1) using microsphere-based Luminex xMAP technology (Luminex, Austin, TX; see [21]), and α- and π-glutathione-S-transferase (α-GST and π-GST) using human ELISA test kits (Argutus Medical, Dublin, Ireland; see [22]).

The common outcome was need for dialysis or death in 30 days. In the development cohort the sensitivity of Functional-AKI was determined. The threshold concentration of the structural biomarker, urinary NGAL, which had the same sensitivity for this outcome was then determined. Patients with urinary NGAL above this threshold were deemed to have Structural-AKI. This threshold was then used to determine the proportions of patients with the outcome in both the development and validation cohorts. Urinary NGAL was chosen for this demonstration because it is the most studied of candidate biomarkers and has been evaluated to be independently associated with mortality and need for dialysis [12].

Results are presented as means ± standard deviation, medians (interquartile range), or n (%). Comparisons were made for normally distributed variables by Students t-test, for non-normally distributed by Mann Whitney U test, and for categorical variables by χ^2^ test. All confidence intervals are 95%. To compare the development and validation cohorts a χ^2^ Goodness-of-fit test was applied. This required a logistic regression model predicting the outcome of [Cohort + AKI + Cohort*AKI], where Cohort equals 0 for the development cohort and 1 for the validation cohort, and the AKI cell equals 0 for no-AKI, 1 for Structural-AKI only, 2 for Functional-AKI only, and 3 for both Structural-AKI and Functional-AKI.

The EARLYARF trial was approved by the multiregional ethics committee of New Zealand (MEC/050020029) and registered under the Australian and New Zealand Clinical Trials Registry (ACTRN012606000032550; http://www.actr.org.au). Screening on entry to ICU was by presumptive consent, followed by written consent from the patient or family.

## Results

The clinical characteristics of the EARLYARF patients have been previously described [16], [17], [23]. Briefly, the 507 patients with available urinary NGAL data were; 39.4% female, 60±18 years of age, 18.1% with Chronic Kidney Disease, 18.9% with sepsis, with a median baseline plasma creatinine of 76 µmol/l (IQR: 60–92 µmol/l), and with a mean APACHE II score of 17.9±6.3. The development cohort had 254 patients and the validation cohort 253 patients. There were no differences in sex, age, weight, Chronic Kidney Disease, baseline plasma creatinine, estimated GFR, APACHE II score, SOFA score, sepsis or NGAL concentrations between the two cohorts (Table 1).

**Table 1:** Demographics of the Development and Validation cohorts.

	Development cohort n = 253	Validation cohort n = 254	p
Female	100 (39.4%)	100 (39.5%)	0.97
Age, years	60.1±17.6	60.0±17.7	0.92
Weight, kg	79.9±18.3	78.4±18.4	0.40
APACHE II	17.7±6.3	18.2±6.6	0.44
SOFA	6.4±2.7	6.1±2.8	0.18
CKD (eGFR<60 ml/min)	44 (17.3%)	48 (19.0%)	0.63
Sepsis	48 (18.9%)	48 (19.0%)	0.98
Baseline plasma creatinine, mmol/L	0.076 (0.060–0.095)	0.075 (0.060–0.091)	0.59
eGFR	87 (67–108)	92 (67–114)	0.47
NGAL, ng/ml	97 (32–400)	83 (32–389)	0.84

Data presented as n(%), means ± sd, or median (interquartile range). APACHE: Acute Physiology and Chronic Health Evaluation; SOFA: Sequential Organ Failure Assessment; CKD: Chronic Kidney Disease; eGFR: estimated Glomerular Filtration Rate using the Modification of Diet in Renal Disease (MDRD) formula.

In the development cohort 110 of 254 (43%) patients had Functional-AKI and 28 of these needed dialysis or died within 30 days. Seventeen patients without Functional-AKI also needed dialysis or died. The sensitivity was thus 62%.

The threshold for urinary NGAL with 62% sensitivity for need for dialysis or death in 30 days was 140 ng/ml. We therefore defined Structural-AKI as NGAL >140 ng/ml. This threshold resulted in 26% (n = 38) of the no Functional-AKI (n = 144) being diagnosed as Structural-AKI (Table 2). Compared with the No-AKI reference group, the relative risk of dialysis or death within 30 days was significantly increased for those with Structural-AKI, Functional-AKI or both (Table 3).

**Table 2:** Patients in the Development cohort versus Validation cohort, n (% of total patients in each cohort).

	Development cohort			Validation cohort		
	No Structural-AKI	Structural-AKI	Total	No Structural-AKI	Structural-AKI	Total
No Functional-AKI	106 (41.7)	38 (15.0)	144 (56.7)	103 (40.7)	41 (16.2)	144 (56.9)
Functional-AKI	45 (17.7)	65 (25.6)	110 (43.3)	46 (18.2)	63 (24.9)	109 (43.1)
Total	151 (59.4)	103 (40.6)	254	149 (58.9)	104 (41.1)	253

Functional-AKI: Plasma creatinine >26.4 µmol/L (0.3 mg/dl) in 48 hours or 50% in 7 days.

Structural-AKI: Urinary NGAL >140 ng/ml.

**Table 3:** Patients having dialysis or death as an outcome, n (% of patients with each diagnosis), and relative risk, RR (95% Confidence interval), in each AKI category in the Development versus Validation cohorts.

	Development cohort		Validation cohort	
Patients with outcome n(%)	No Structural-AKI	Structural-AKI	No Structural-AKI	Structural-AKI
No Functional-AKI	9 (8.5)	8 (21.1)	10 (9.7)	6 (14.6)
Functional-AKI	8 (17.8)	20 (30.7)	6 (13.0)	19 (30.2)
**Relative Risk of outcome (95% Confidence interval)**				
No Functional-AKI	1 (referent)	2.48 (1.94 to 2.81)	1 (referent)	1.51 (1.26 to 1.62)
Functional-AKI	2.09 (1.70 to 2.31)	3.62 (2.95 to 4.16)	1.34 (1.16 to 1.42)	3.11 (2.53 to 3.55)

Functional-AKI: Plasma creatinine >26.4 µmol/L (0.3 mg/dl) in 48 hours or 50% in 7 days.

Structural-AKI: Urinary NGAL >140 ng/ml.

Outcome: Need for dialysis or death within 30 days.

The validation cohort was similar to the development cohort. There was no difference in the proportions of Structural-AKI (urinary NGAL>140 ng/ml) and Functional-AKI to the development cohort (p = 0.76, Table 2) or of those who needed dialysis or died (p = 0.56, Table 3). The relative risk of dialysis or death was greatest in the cohort with both Structural-AKI and Functional-AKI in both development and validation cohorts (Table 3). The risk of death or dialysis in the validation cohort was not different from that of the development cohort (p = 0.87). The sensitivity for AKI (either Structural or Functional) was 76% (31 died or needed dialysis with AKI, 10 without).

This method of determining thresholds may be extended to determine biomarker thresholds for each severity stage of Functional-AKI. Using the entire cohort we determined that the threshold equivalents for KDIGO Functional-AKI for NGAL were for: Stage 1, 140 ng/ml; Stage 2, 438 ng/ml; Stage 3, 2710 ng/ml. These are presented in Table 4 along with the thresholds for urinary alkaline phosphatase, γ-glutamyl transpeptidase, cystatin C, interleukin-18, kidney injury molecule-1, and α- and π-glutathione-S-transferase.

**Table 4:** Structural-AKI thresholds for severity stages based on equivalent sensitivity (62%) to Functional-AKI.

Functional-AKI stage	NGAL	AP	GGT	Cystatin C	IL-18	KIM1	α-GST	π-GST
	ng/ml	U/L	U/L	mg/L	pg/ml	pg/ml	μg/L	μg/L
**1.** ≥26.4 µmol/L in 48h or ≥50% in 7d	140	11.2	128	0.95	118	1850	5.13	14.6
**2.** ≥100% in 7d	438	28.2	254	7.77	735	6530	31.1	59.3
**3.** ≥200% or increase to ≥353.6 µmol/L in 7d	2710	50.2	452	17.0	2330	9760	82.1	141

## Discussion

Literature thresholds for urinary NGAL diagnosis of AKI vary with cause and context of AKI and range from 72 ng/ml in children after cardio-pulmonary bypass surgery [24] to 680 ng/ml in adults after cardio-pulmonary bypass surgery [25]; Using these extremes in the two studies cited, the demonstrated sensitivities for the subsequent creatinine-based diagnosis of AKI ranged respectively from 42% to 62.5%. In our illustration, the threshold selected in a heterogenous ICU population using a sensitivity of 62% was also based on a currently accepted consensus definition of Functional-AKI. While this hypothesis clearly requires external validation in much larger datasets, the data appear robust compared with literature values and allows determination of subset relative risk. The present dataset contained only sufficient patients for a power of 73% at an α of 0.05 for comparison of the validation cohort to the development cohort. A data set of 750 patients would be needed to extend this to a power of 90%. Ideally, disaggregated data from multiple data sets would be used to determined thresholds for each biomarker.

As expected, the use of injury biomarkers of AKI resulted in more people being diagnosed with AKI. However, by linking the diagnosis of Functional-AKI with Structural-AKI through sensitivity to the same hard outcomes, clinicians can be confident in the clinical implications of the detected increase in injury biomarker. The technique will also enable the use of multiple biomarkers, each of which potentially reflect a different mechanism and time course of injury. The demonstration, that a low urinary biomarker (NGAL) concentration reduces risk, illustrates additional potential benefits from this strategy. We consider it important that when applying this methodology to determine thresholds the biomarker reflects injury specific to the kidney and not some other disease, such as sepsis that may also be independently related to mortality. This may mean different thresholds in different cohorts, such as for urinary Cystatin C [26] or NGAL [27], [28] in sepsis, as well as recognising that the kidney injury “signal” may be drowned out by other sources of the biomarker in some circumstances, thus rendering the biomarker not diagnostic of Structural-AKI.

We proposed sensitivity of Functional-AKI for determining thresholds because early stage AKI management choices are low-risk (see KDIGO guidelines, figure four [6]) and relationship with outcome is well established and similarly low risk. Alternatives include defining the thresholds using specificity, a predetermined sensitivity or specificity, or optimisation for both sensitivity and specificity. Specificity should be considered if interventions are high risk. In our illustration, the specificity of Functional-AKI was low (60.8%) resulting in threshold for NGAL of 127 ng/ml, similar to that determined using sensitivity. Choosing a pre-specified higher specificity will increase thresholds of both Functional-AKI and Structural-AKI, whereas choosing a higher sensitivity will produce decreases. As the definition of Functional-AKI has been determined by consensus and based on evidence that an increase in creatinine of as little as 0.3 mg/dl is associated with poor outcomes [14], we do not propose that a pre-specified specificity or sensitivity be used. It is highly unlikely that a biomarker threshold for Structural-AKI could be chosen with the identical sensitivity and specificity as Functional-AKI. An alternative strategy is to determine the nearest point on the biomarker receiver operator characteristic (ROC) curve (Sensitivity verse 1-Specificity) that is closest to the point on the creatinine ROC with the sensitivity and 1-specificity of Functional-AKI. While this avoids having to choose either sensitivity or specificity it would introduce an imbalance between the performance of Functional-AKI and Structural-AKI in terms of relative risk for mortality and dialysis need.

It is a point of debate whether mortality alone or mortality and dialysis need together should be the outcome measure used. We proposed the combined outcome of need for dialysis and mortality because of clinical relevance and the association of both Functional-AKI and kidney injury biomarkers with both dialysis and mortality. A rapidly increasing creatinine is clearly an indication for dialysis. Multiple biomarkers originating in the kidney have been shown to be associated with dialysis need [12], [17]. Functional-AKI is known to be associated with mortality, indeed the threshold of an increased creatinine of 0.3 mg/dl was based on the association with mortality [14] and multiple kidney injury biomarkers have been shown to be associated with mortality [12], [17]. For biomarkers that are associated with disease states other than kidney injury, it may be that there is some bias introduced because of the biomarker reflecting other illness in addition to kidney injury. This means that some of the mortality associated with the biomarker may not be because of the severity of kidney injury. These specific circumstances need to be identified and, if possible, the bias needs to be quantified, before application of the proposed methodology.

**Figure 1:**
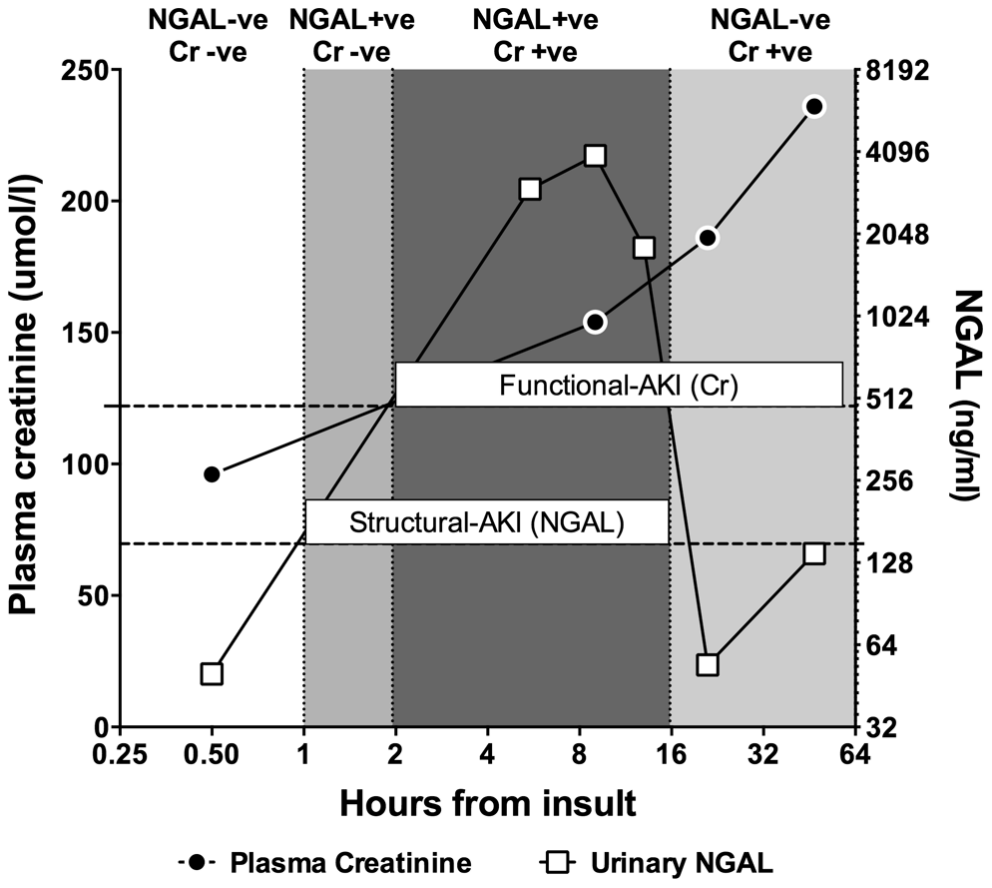
Illustrative biomarker time course following a cardiac arrest. Baseline creatinine was 96 µmol/l. Horizontal dotted lines represent the thresholds for Functional-AKI (26.4 µmol/l increase over baseline) and Structural-AKI (140 ng/ml). If the diagnosis of Structural-AKI and Functional-AKI were to be made at only one time point then the patient would be initially negative for both classifications before becoming positive for Structural-AKI for a short period whilst remaining negative for Functional-AKI. From 2 to 16 hours the patients is positive for both Structural and Functional-AKI before becoming negative again for Structural-AKI.

As suggested by Siew et al there is a need to examine large datasets to assess the agreement or disagreement with creatinine data [29]. Important for the development of thresholds are datasets, which contain sample biomarker concentrations at multiple time points. Utilising the maximum concentration rather than concentration at any one time point, as we have done in this illustration, will minimise miss-classifying patients merely because of the timing of the biomarker sample. Figure 1 illustrates how one patient may be classified to each of the four possible diagnostic classes if classification is made only on the basis of one sample at a single time point. When viewed in hindsight and with the benefit of all time points, the patient clearly had both a loss of GFR and structural injury caused by the cardiac arrest. The type of AKI diagnosed (Structural, Functional or both) is therefore merely a function of the timing of sampling, given the differences in temporal profiles of serum creatinine and urinary NGAL. This also highlights that if there is a clinical need for identifying both functional change and injury there is a need for serial sampling. Our method illustrates how these datasets may be used to establish injury biomarker thresholds. This will allow comparison of thresholds for different aetiologies of AKI and in different patient groups, for example those with and without sepsis.

In conclusion, sensitivity to need for dialysis and death can be used to link and give equal weight to functional or structural biomarker-based definitions of AKI. This hypothesis awaits validation in large multicentre datasets.
